# Synthesis of Substituted 1,4-Diazepines and 1,5-Benzodiazepines Using an Efficient Heteropolyacid-Catalyzed Procedure

**DOI:** 10.3390/molecules16010092

**Published:** 2010-12-28

**Authors:** Rachedine Kaoua, Norah Bennamane, Saliha Bakhta, Sihame Benadji, Cherifa Rabia, Bellara Nedjar-Kolli

**Affiliations:** 1 Laboratory of Applied Organic Chemistry, Houari Boumediene University of Sciences and Technology, BP 31, El-Alia, Bab-Ezzouar, 16111, Algiers, Algeria; 2 Center of Research in Physical and Chemical Analysis CRAPC, BP 248, Algiers RP 16004, Algiers, Algeria; 3 Laboratory of Natural Gas, Houari Boumediene University of Sciences and Technology, BP 31, El-Alia, Bab-Ezzouar 16111, Algiers, Algeria

**Keywords:** 1,4-diazepine, 1,5-benzodiazepine, dehydroacetic acid, 1,3-aminomethyl propane, orthophenylene diamine, heteropolyacids

## Abstract

An efficient and improved procedure for the synthesis of 1,4-diazepine and 1,5-benzodiazepine derivatives via the reaction of ketimine intermediates with aldehydes in the presence of Keggin-type heteropolyacids (HPAs) was developed. High yields and short reaction times were obtained for both electron-releasing and electron-withdrawing substituted 1,4-diazepine and 1,5-benzodiazepines derivatives.

## 1. Introduction 

Diazepines and benzodiazepines have various therapeutic applications. Many members of the diazepine family are widely used as anticonvulsants, antianxiolitics, analgesics, sedatives, antidepressives and hypnotic agents [1,2,3,4]. Benzodiazepine derivatives are used as dyes for acrylic fibers [5]. In addition, benzodiazepines are valuable intermediates for the synthesis of fused ring compounds such as triazolo-, oxadiazolo-, oxazino-, and furanobenzodiazepines [6,7,8,9,10,11]. 

Due to their wide range of pharmacological activity and industrial applications, the development of mild and efficient protocols for their preparation continues to be a challenging endeavor in the synthetic organic chemistry [12,13,14,15,16,17,18,19,20,21,22,23,24,25,26,27,28]. The common procedure for the synthesis of these compounds is a one pot condensation between *o*-phenylenediamines and carbonyl compounds [29]. However, a large number of the modified methods reported in the literature, suffer from several drawbacks such as the use of a large amount of catalysts, unsatisfactory product yields and critical product isolation procedures. These disadvantages require a development of an efficient and practically useful process of preparation [30].

In recent years, the use of polyoxometalates as catalysts in homogeneous and heterogeneous systems has attracted great interest in organic synthesis because their acidic and redox properties can be controlled at the molecular or atomic level and they also offer economical and environmental benefits [31,32,33,34,35].

Among the different classes of polyoxometalates, the Keggin-type heteropolyacids (HPAs) offer a strong option for efficient and cleaner processing compared to polluting and corrosive liquid acid catalysts [36]. They have the particularity of possessing multi-functional properties as strong Brønsted acidity and strong redox power. Their use allows eductions of costs and waste generation through reuse and recycling [37,38]. 

We report here for the first time the synthesis of 1,4-diazepines **4** from enaminones **2** in the presence of both CF_3_COOH and Keggin type HPA catalysts. 1,5-Benzodiazepines **5** have also been prepared, again in the presence of the HPA catalysts [29]. 

## 2. Results and Discussion 

**Scheme 1:**
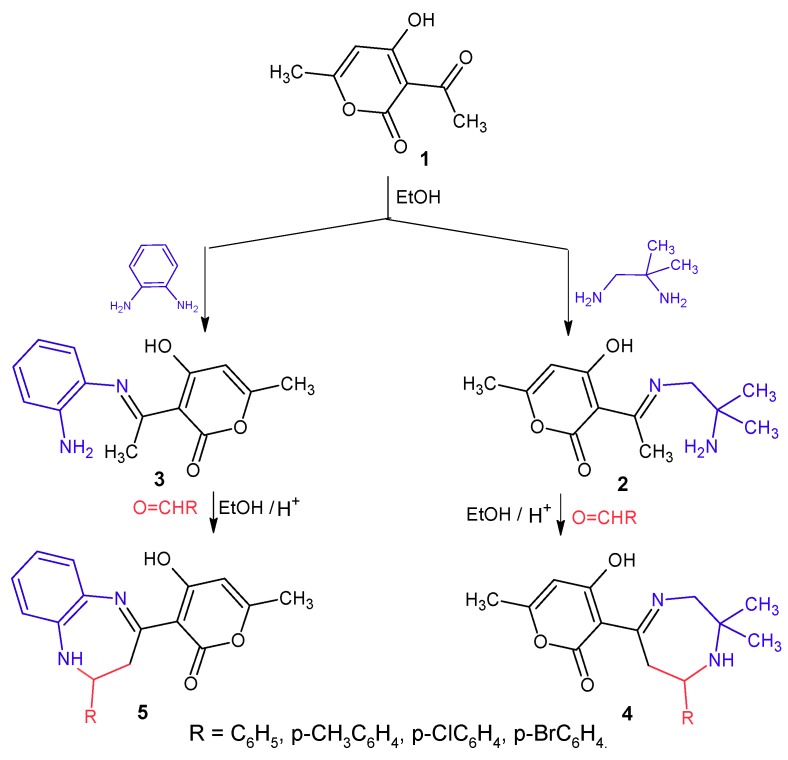
Synthesis of 1,4-benzodiazepines **4** and 1,5- benzodiazepines **5**.

Previously, we have synthesized 1,5-benzodiazepine **5** products [29] from the reaction between product **3** and aromatic aldehydes in the presence of catalytic amounts of trifluroacetic acid in ethanol under reflux conditions. In continuation of this work on the synthesis of diazepine rings, we have now prepared a series of novel 1,4-diazepines **4** from derivatives **2** following to the same procedure (Scheme 1). The results (Table 1) show that the reaction is slow with unsatisfactory yields. 

Due to strong Brønsted acidity and high oxidative power of Keggin type HPAs, that give them a bifunctional character, we decided to test the following series of HPAs in the preparation of derivatives **4** and **5**: H_3_PW_12_O_40_ and H_3+x_PMo_12-x_V_x_O_40_, with x = 0-3, as catalysts. The obtained results are shown in Table 2.

The catalysts were used with equimolar amounts of reactants in refluxing ethanol; the reaction proceeded smoothly, affording the products in good to excellent yields. The results indicate that the catalytic activity depends on the composition of the catalyst. The substitution of one to three molybdenum atoms by vanadium atoms decreases the reaction time and increases the product yields. This is can be attributed to the character more oxidative and less acidic compared to those of H_4_PMo_11_VO_40_, H_3_PMo_12_O_40_ and H_3_PW_12_O_40_ [31,35,38]. As shown in Table 2, the order of efficiency follows the sequence: H_5_PMo_10_V_2_O_40_> H_6_PMo_9_V_3_O_40_> H_4_PMo_11_VO_40_ > H_3_PMo_12_O_40_ > H_3_PW_12_O_40_. Since the best results in term of yield and reaction time have been achieved with H_5_PMo_10_V_2_O_40_, this was selected for its better catalytic performance for the synthesis of diazepine rings.

**Scheme 2:**
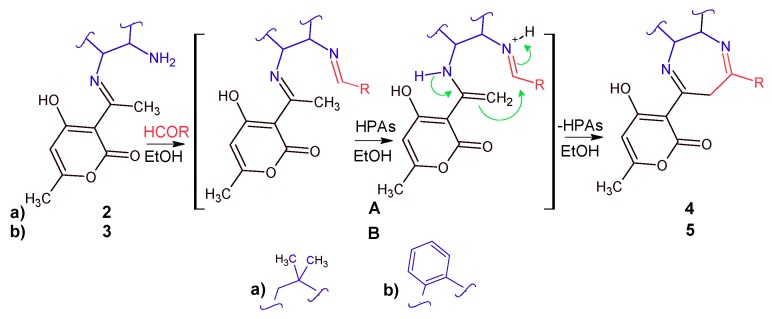
Synthetic route to compounds **4** and **5**.

To optimize the reaction, temperature and molar ratio of H_5_PMo_10_V_2_O_40_ catalyst and reactants were checked. No reaction was detected at room temperature. Intramolecular cyclization of **2** or **3** via respectively intermediate **A** or **B** (Scheme 2) under reflux conditions in the presence of only 0.1 % of H_5_PMo_10_V_2_O_40_ catalyst is enough to drive the reaction forward; higher amounts of catalysts did not improve the results. 

It is difficult to clarify how HPAs acts as a catalyst in this reaction. On the basis of a previously reported mechanism [29], it was suggested that the formation time of derivatives **5** depends on the nature of the substituent R on the aldehyde ring. Formation of **5** is particularly rapid when R is a strong donor group, whereas a withdrawing substituent inhibits the formation of compounds **5**. Yields are also affected by electronic effects and they significantly increased by using an excess of aldehyde (Table 1). Similarly, intermediate **A** undergoes intramolecular cyclization under acidic conditions, according to the described conditions [29] leading to products **4**. Yields and reaction times are very comparable to those obtained with intermediate **B**. These results can be explained by the nature of substituent R as shown in Table 1. When the optimized conditions (in presence of HPAs) are applied to substrates, high yields of **4** and **5** are obtained. These results show that the electronic nature of the substituent seems not to have a great effect on the reaction yields and that both acidic and redox properties of the heteropolyacids are involved during the synthesis.

The nature and the composition of the polyanion have an important effect on the catalytic properties of the HPAs. However, it is very difficult to clarify the difference between catalytic behaviour of these systems in this reaction. We believe that there is a complex relationship between the activity, the chemical composition and the position of the vanadium atoms in the Keggin polyanions. 

## 3. Experimental 

### 3.1. General 

Melting points were measured on a Buchi 512 apparatus and were uncorrected. FTIR spectra were taken in Nujol on a Perkin –Elmer spectrometer. The ^1^H-NMR spectra (250 and 300 MHz) and ^13^C- NMR (63 MHz) were run on a Bruker spectrometer in CDCl_3_ using tetramethylsilane as internal standard. The impact ionisation (IE) mass spectra were recorded on a Nermag R-10-10C at 70 ev. Chemicals were purchased from Aldrich and Fluka. Compounds **2** and **3** were prepared according to the literature [39]. All the products were identified by comparison of analytical data (mp, NMR) with those reported (in the case of derivatives **5**) or with authentic samples prepared by the conventional method using CF_3_COOH as catalyst (in the case of compounds **4**).

### 3.2. Catalyst preparation

Phosphomolybdic and phosphotungstic acid samples, H_3_PMo_12_O_40_ and H_3_PW_12_O_40_ were obtained according to the method of Tsigdinos [40] by heating MoO_3_ (WO_3_) with a solution of diluted H_3_PO_4_. Synthesis of mono-, di- and tri-vanadium substituted phosphomolybdic acids, H_3+x_[PMo_12−x_V_x_O_40_] (*x* = 1–3) was carried out according to the procedure of Tsigdinos and Hallada [41] by acidifying with concentrated H_2_SO_4_ an appropriate mixture solutions of Na_2_HPO_4_, Na_2_MoO_4_ and NaVO_3_ in appropriate molar ratio. The HPAs formed were extracted with diethyl ether.

### 3.3. General procedure for the heteropolyacid-Keggin acid catalyzed synthesis of derivatives ***4*** and ***5***

A mixture of **2** or **3** (10 mmol) and aldehyde (10 mmol) in presence of Keggin acid catalyst (1% mmol) dissolved in ethanol (15 mL) was refluxed with stirring for the indicated time (see Table 2). The reaction was followed by TLC. When the reaction time was over, the organic solution was concentrated in vacuum. Then the compounds were filtered and washed twice with ethanol (2 × 10 mL). The catalyst was recovered after evaporating of ethanol. 

*3-(2,2-Dimethyl-7-phenyl-2,3,6,7-tetrahydro-1H-1,4-diazepin-5-yl)-4-hydroxy-6-methyl-2H-pyran-2-one* (**4a**).Yellow solid, m.p. 186 ºC; ^1^H-NMR (CDCl_3_, 250 MHz): δ 1.45 (s, 3H, CH_3_), 1.48 (s, 3H, CH_3_), 2.13 (s, 3H, CH_3_), 3.58 (s, 2H, CH_2_-N=), 4.32 (t, 1H, *J* = 9 Hz, CH-Ar), 4.75 (dd, 2H, *J* = 9, 2 Hz, CH_2_-CH), 5.85 (s, 1H, =CH), 7.16-7.37 (m, 5H, H-Ar), 7.52 (s, 1H, NH), 13.80 (s, 1H, OH); ^13^C-NMR (CDCl_3_): δ 20, 22, 30, 52, 53, 55, 62, 96, 108, 128, 129, 132, 143, 163, 164, 179, 184; MS *m/z* (%): 326 ([M^·^]^+^, 6), 235 (19), 181 (60), 151 (27), 136 (14), 43 (37).

*3-[2,2-Dimethyl-7-(4-methylphenyl)-3,6-dihydro-2H-1,4-diazepin-5-yl]-4-hydroxy-6-methyl-2H-pyran-2-one* (**4b**).Yellow solid, m.p. 220 ºC; ^1^H-NMR (CDCl_3_, 250 MHz): δ 1.37 (s, 3H, CH_3_), 1.38 (s, 3H, CH_3_), 2.14 (s, 3H, CH_3_), 2.24 (s, 3H, CH_3_), 3.54 (s, 2H, CH_2_-N=), 4.17 (t, 1H, *J* = 9 Hz, CH-Ar), 4.70 (dd, 2H, *J* = 9, 2 Hz, CH_2_-CH), 5.75 (s, 1H, =CH), 7.00-7.10 (d, 2H, *J* = 8.3 Hz, H-Ar), 7.10-7.20 (d, 2H, *J* = 8.1 Hz, H-Ar), 7.80 (s, 1H, NH), 14.03 (s, 1H, OH); ^13^C-NMR (CDCl_3_): δ 20, 21, 32, 51, 53, 55, 61, 95, 108, 128 - 129, 134, 146, 163, 164, 178, 18; MS *m/z* (%): 340 ([M^·^]^+^, 2), 235 (16), 181 (40), 151 (4), 136 (1), 43 (70).

*3-[7-(4-Chlorophenyl)-2,2-dimethyl-2,3,6,7-tetrahydro-1H-1,4-diazepin-5-yl]-4-hydroxy-6-methyl-2H-pyran-2-one* (**4c**).Yellow solid, m.p. 223 ºC; ^1^H-NMR (CDCl_3_, 250 MHz): δ 1.38 (s, 3H, CH_3_), 1.39 (s, 3H, CH_3_), 2.15 (s, 3H, CH_3_), 3.65 (s, 2H, CH_2_-N=), 4.39 (t, 1H, *J* = 9 Hz, CH-Ar), 4.70 (dd, 2H, CH_2_, *J* = 9, 2 Hz, CH_2_-CH), 5.90 (s, 1H, =CH), 7.20-7.25 (m, 2H, H-Ar), 7.25-7.30 (m, 2H, H-Ar), 7.96 (s, 1H, NH), 13.82 (s, 1H, OH); ^13^C-NMR (CDCl_3_): δ 19, 20, 30, 50, 54, 55, 63, 96, 105, 129, 130, 133, 136, 165, 166, 177, 182; MS *m/z* (%): 360 ([M^·^]^+^, 24), 235 (14), 181 (100), 151 (8), 136 (8), 43 (28).

*3-[7-(4-bromophenyl)-2,2-dimethyl-2,3,6,7-tetrahydro-1H-1,4-diazepin-5-yl]-4-hydroxy-6-methyl-2H-pyran-2-one* (**4d**). Yellow solid, m.p. 245 ºC; ^1^H-NMR (CDCl_3_, 250 MHz): δ 1.44 (s, 3H, CH_3_), 1.47 (s, 3H, CH_3_), 2.15 (s, 3H, CH_3_), 3.62 (s, 2H, CH_2_-N=), 4.36 (t, 1H, *J* = 9 Hz, CH-Ar), 4.70 (dd, 2H, *J* = 9, 2 Hz, CH_2_-CH), 5.90 (s, 1H, =CH), 7.10-7.20 (m, 2H, H-Ar), 7.35-7.45 (m, 2H, H-Ar), 7.99 (s, 1H, NH), 13.79 (s, 1H, OH); ^13^C-NMR (CDCl_3_): δ 19, 20, 34, 53, 54, 56, 60, 95, 107, 130, 131, 135, 145, 163, 165, 178, 181; MS *m/z* (%): 371 ([M^·^]^+^, 0.5), 235 (18), 181 (20), 151 (3), 136 (5), 43 (66).

## 4. Conclusions 

We present here the synthesis of a new series of 1,4-benzodiazepine derivatives **4** using two different catalyst classes, a conventional catalyst, CF_3_COOH, and HPAs. The results have shown the superior catalytic efficiency of the heteropolyacids. The principal features of the methodology using HPAs as catalyst are high-yields and shorter reaction times for both electron-releasing and electron-withdrawing substituted derivatives **4** and **5**, substances with potentially interesting biological and medicinal properties.

## Figures and Tables

**Table 1:** Synthesis of 1,4-diazepine and 1,5-benzodiazepine derivatives using CF_3_COOH as catalyst under reflux conditions.

Compounds	R	Ratio of aldehyde	CF_3_COOH Times (min) / Yields (%)
**4a**	C_6_H_5_	1.5	420/60
**4b**	*p*-CH_3_C_6_H_5_	1.5	420/55
**4c**	*p*-ClC_6_H_5_	2.5	360/64
**4d**	*p*-BrC_6_H_5_	2.5	360/69
**5a**	C_6_H_5_	1.5	360/79
**5b**	*p*-CH_3_C_6_H_5_	1.5	420/83
**5c**	*p*-ClC_6_H_5_	3	720/64
**5d**	*p*-BrC_6_H_5_	3	720/74

**Table 2:** Synthesis of 1,4-diazepine and 1,5-benzodiazepine derivatives using equimolar reactants in presence of various heteropolyacids under refluxing conditions.

Compounds	R	H_3_PW_12_O_40_	H_3_PMo_12_O_40_	H_4_PMo_11_VO_40_	H_5_PMo_10_V_2_O_40_	H_5_PMo_9_V_3_O_40_
		Times (min) / Yields (%)				
**4a**	C_6_H_5_	600/72	300/69	180/70	30/85	60/83
**4b**	*p*-CH_3_C_6_H_5_	660/75	300/79	180/77	30/80	60/83
**4c**	*p*-ClC_6_H_5_	640/69	300/73	180/73	30/78	60/74
**4d**	*p*-BrC_6_H_5_	600/75	300/71	180/72	30/75	60/77
**5a**	C_6_H_5_	420/87	220/79	120/85	15/90	40/78
**5b**	*p*-CH_3_C_6_H_5_	390/82	220/83	120/82	15/88	40/89
**5c**	*p*-ClC_6_H_5_	360/70	220/77	120/75	15/93	40/87
**5d**	*p*-BrC_6_H_5_	360/76	220/73	120/74	15/91	40/94
